# Acid-assisted polysaccharides extracted from *Asparagus cochinchinensis* protect against Alzheimer’s disease by regulating the microbiota-gut-brain axis

**DOI:** 10.3389/fnut.2024.1496306

**Published:** 2024-12-18

**Authors:** Ruixue Li, Hui Wang, Qinjian Wang, Zhiqiang Zhang, Li Wang

**Affiliations:** ^1^College of Pharmacy, Henan University of Chinese Medicine, Zhengzhou, Henan, China; ^2^Henan Provincial Hospital of Traditional Chinese Medicine, Henan University of Chinese Medicine, Zhengzhou, Henan, China; ^3^Department of Traditional Chinese Medicine, Henan Agricultural University, Zhengzhou, China

**Keywords:** polysaccharide, *Asparagus cochinchinensis*, microbiota-gut-brain, identification of structure, gut microbiota

## Abstract

In this study, an acid-assisted extraction strategy was used to extract a novel polysaccharide (ACP) from *Asparagus cochinchinensis,* after which this polysaccharide was purified and subjected to extensive characterization. ACP was determined to have an average molecular weight of 15,580 Da in structural characterization studies, and it was found to primarily consist of glucose, galactose, L-fucose, and fructose at an 82.14:12.23:2.61:2.49 ratio. Trace amounts of xylose, arabinose, and rhamnose were also detected in ACP preparations at a 0.48:0.04:0.02 ratio. GC–MS analyses identified eight different sugar linkages within ACP, including Glc*p*-(1→, →2)-Glc*p*-(1→, →6)-Glc*p*-(1→, →4)-Glc*p*-(1→, →3, 4)-Glc*p*-(1→, →2,4) -Gal*p*-(1→, →4,6)-Gal*p*-(1→, and →3,4,6)-Gal*p*-(1 → linkages present at 23.70:1.30:3.55:50.77:6.91:1.10:11.50:1.18 molar percent ratios. One-dimensional NMR, two-dimensional NMR, and methylation analyses ultimately revealed that the polysaccharide is mainly composed of →4)-*β*-D-Glc*p*-(1 → and a small amount→4,6)-*α*-D-Gal*p*-(1 → and →3,4)-*α*-D-Glc*p*-(1 → and so on. Branched chain is mainly composed of α-D-Glc*p*-(1 → 4)-*β*-D-Glc*p*-(1 → connected to the sugar residues α-D-Glc*p*-(1 → 4)-β-D-Glc*p*-(1 → O-4 position or sugar residues of α-D-Glc*p*-(1 → 4)-β-D-Glc*p*-(1 → O-3 position. ACP treatment in SAMP8 mice was associated with reductions in oxidative stress and brain pathology together with enhanced cognitive function. ACP treated SAMP8 mice also presented with increases in *Bacteroidota* abundance and reduced *Firmicutes, Patescibacteria, Actinobacteriota,* and *Campilobacterota* abundance. Thus, ACP can prevent Alzheimer’s disease by modulating the microbe-gut-brain axis.

## 1 Introduction

Alzheimer’s disease (AD) is a progressive neurogenerative disease that is currently regarded as being irreversible (1, 2). While the pathogenesis of AD is incompletely understood, the pathological hallmarks of this disease include the formation of senile plaques comprised of *β*-amyloid (Aβ) deposits and neurofibrillary tangles together with chronic inflammatory responses that entail the activation and proliferation of glial cells, dysfunctional synaptic activity, and the degeneration and death of neurons (3, 4). Inflammatory responses arise in the tissue surrounding Aβ deposits, and cerebral microvascular Aβ deposition has repeatedly been established as a driver of neuroinflammation in AD patients (5). The astrocytic and microglial activation evident in AD patients is associated with the release of a range of pro-inflammatory cytokines and chemokines that further propagate the neuroinflammatory cascade (6, 7). The high levels of inflammatory mediators and complement cascade activity within AD patient brain tissue provide strong support for the pathogenic role of inflammation in this disease. The induction of this complex neuroinflammatory cascade is believed to be largely mediated by adhesion molecules and chemokine signaling. In this study, the senescence-accelerated mouse prone 8 (SAMP8) model was leveraged to better study the inflammatory microbiota-gut-brain *axis* and its association with AD.

A growing body of evidence suggests that the gastrointestinal microflora is important not only for gut homeostasis, but also the function of distant organs such as the brain (8, 9). A complex bidirectional communication system referred to as the microbiota-gut-brain axis has been proposed to explain this regulatory relationship (10). Through this microbiota-gut-brain axis, the dysbiosis of the gut microflora can impact psychiatric symptoms and cognitive function (11), while also modulating the homeostatic balance of immune activity within the brain, potentially contributing to the initiation or progression of age-related neurodegenerative conditions including AD, multiple sclerosis, and Parkinson’s disease (12, 13). Mechanisms whereby gut microbes can reportedly affect the progression of AD include the differential regulation of neuroinflammatory activity, oxidative stress, Aβ deposition, and other factors linked to neuronal death (14, 15). The most recent research evidence supports the ability of the gastrointestinal microflora to help delay aging-related activity and alleviate cognitive impairment in part via the mitigation of oxidative stress (16). The precise mechanisms whereby these gut microbes can counteract aging-related processes through this microbiota-gut-brain axis, however, remain to be firmly established (17).

Traditional Chinese medicine (TCM) strategies have long been used to prevent or treat neurodegenerative diseases, and interest in their use has risen substantially in recent years. *Asparagus cochinchinensis* (AC) (Lour.) Merr. (Asparagi radix), also known as Tiandong, is an herb that is widely used in TCM practices (18). A member of the Liliaceae family, AC is used to nourish Yin, clear the lungs, moisten dryness, and promote the secretion of fluid under TCM theory (19). AC is a perennial plant found growing in China, Korea, and Japan, and it has been applied in TCM practices to treat conditions including coughs, fevers, inflammatory diseases, renal diseases, brain diseases, and breast cancer (20). Polysaccharides are natural polymers consisting of greater than 10 monosaccharide units’ joints by glycosidic linkages in linear or branching chains that can have very large molecular weights (21–23). Naturally derived polysaccharides can have a range of immunomodulatory effects in clinical settings (24). Polysaccharides can also influence developmental processes, exerting a diverse range of antioxidant (25, 26), anti-inflammatory, hypoglycemic, antithrombotic, anticoagulant, antiviral, antitumor, and anti-complement activities (27–29). The precise processing technologies employed for AC can impact its functional, physicochemical, and microstructural properties. While a variety of plant polysaccharide extraction methods have been described (30, 31), only an aqueous extraction-based strategy has thus far been reported for the isolation of AC polysaccharides (ACPs) (18, 32). Moreover, SAMP8 mice are a subline of the SAM model first generated in the 1970s at Kyoto University that experience pronounced memory and learning impairments that worsen with progressive aging, making them ideally suited to studies of age-related disease. Importantly, these mice present with the overproduction of Aβ such that they are regarded as a model of early AD pathology (33). In this study, an acid extraction strategy was used to aid in ACP extraction, after which gas chromatography–mass spectrometry (GC–MS), gel permeation chromatography (GPC), high-performance anion-exchange chromatography (HPAEC), Fourier-transform infrared spectroscopy (FT-IR), and nuclear magnetic resonance (NMR) spectroscopy were employed for the structural characterization of the isolated polysaccharides. After characterizing ACP preparations, the impact of ACP administration on neuroinflammation and Aβ deposition within the brains of rapidly aging SAMP8 mice was evaluated and the impact of ACP administration on oxidative stress and inflammation in the brains of SAMP8 mice was assessed based on the levels of inflammatory mediators and Aβ present therein.

## 2 Materials and methods

### 2.1 Chemicals and reagents

*Asparagus cochinchinensis* was obtained from Guangxi (China). All reagents were from Sinopharm Chemical Reagent Co., Ltd. (Shanghai, China) and of analytical grade unless otherwise indicated.

### 2.2 AC preparation

After drying at 45°C, AC roots were pulverized with a mortar in the laboratory and passed through a 40-mesh screen to generate a fine powder from which ACPs were subsequently extracted.

### 2.3 Acid-assisted extraction

While certain polysaccharides can be extracted through the use of diluted acid solutions, others, particularly acidic polysaccharides or those polysaccharides that contain uronic acid, can be extracted more readily using alkaline solutions. For this study, an acid-assisted extraction procedure was employed. Briefly, 5.0 g of AC powder was refluxed twice with 85 mL of 0.1 moL/L HCl solution for 2.5 h at 85°C, after which these extraction solutions were neutralized, concentrated, and precipitated using a final 75% ethanol concentration for 12 h at 4°C. Next, 80 mL of distilled water was used to suspend 6 g of this crude polysaccharide preparation, followed by centrifugation (10 min, 10,000 xg) with subsequent separation of the supernatant using a DEAE Sepharose Fast Flow column and elution using water and solutions containing various concentrations of NaCl (0, 0.1, and 0.3 mol/L). Different eluates were concentrated, dialyzed, and lyophilized to generate the ACP-A, ACP-B, and ACP-C fractions, of which 100 mg of ACP-B was dissolved with 4 mL of 0.1 mol/L NaCl followed by centrifugation (10 min, 10,000 xg). The supernatant fraction was then separated with a Superdex™200 column and eluted using 0.1 mol/L NaCl to yield the major polysaccharide (ACP).

### 2.4 ACP physicochemical characterization and structural analyses

#### 2.4.1 ACP physicochemical characterization

The respective phenol-sulfuric acid, bovine serum protein-Coomassie bright blue, and Folin–Ciocalteu methods were used for analyses of total carbohydrate, protein, and phenolic content (32).

#### 2.4.2 Molecular weight and monosaccharide composition analyses

ACP molecular weight values were measured via GPC with a Sugar KS 805 column (50 × 8.0 mm, Shodex, Tokyo, Japan) and a differential refractive index detector, using 0.02 M sodium phosphate buffer (pH 6.8) as an eluent at a 1 mL/min flow rate with a column temperature of 30°C. A 30 μL injection volume was used, and a range of dextran standards (4,320, 12,100, 73,800, 121,000, 289,000, and 491,000 Da) were used to generate a calibration curve for molecular weight.

ACP monosaccharide composition was analyzed with an ion exchange chromatography-pulse amperometric detection system (IEC-PAD, Thermo Fisher, United States) (34). Briefly, 5 mg ACP samples were hydrolyzed for 24 h with 3 mL of trifluoroacetic acid at 100°C, followed by the addition of 5 mL of methanol three times. Samples were evaporated until dry, after which the residue was dissolved in 10 mL of 1 M NaOH. Samples were next filtered and injected into an ion chromatography system (Dionex, ICS-5000+, United States) using an AS-AP autosampler and a Carbopac PA-20 column (3 × 150 mm, Dionex). Monosaccharides present in ACP were determined with reference to 9 benchmark compounds (fucose, arabinose, galactose, glucose, xylose, mannose, fructose, galacturonic acid, and glucuronic acid).

#### 2.4.3 FT-IR analysis

An FT-IR instrument (Vertex 70, Bruker, Germany) was used for FT-IR analyses with a 4,000–400 cm^−1^ spectral range, measuring sample transmittance for KBr pellets with a width of 7 mm.

#### 2.4.4 NMR spectroscopy

ACP sample (50 mg) were dried overnight under vacuum, followed by resuspension in 0.6 mL of D_2_O. These samples were then analyzed to generate ^1^H NMR, ^13^C NMR, DEPT 135 NMR, 2D ^1^H-^1^H COSY, HSQC, and HMBC NMR spectra at 25°C with a Bruker Avance III 600 spectrometer (Bruker, Germany) and a PABBO probe (5 mm, BB/19F-1H/D, Z-GRD). As an internal standard, acetone was selected, with respective 31.45 and 2.225 ppm shifts for ^13^C and ^1^H NMR relative to acetone. Standard Bruker software and MestNova were used to process all resultant data.

#### 2.4.5 Scanning electron microscopy

ACP surface morphology was assessed with a Quanta 250 FEG (FEI, America) SEM instrument. Briefly, samples were coated with a thick layer of gold, placed onto the substrate, and imaged at 10 kV with 1K–100K magnification under high vacuum.

### 2.5 Animal studies

In total, 24 male SAMP8 mice and 6 senescence-resistant controls (SAMR1, NC) with a body weight of ~25.0 g were obtained from Beijing Weitong Lihua Laboratory Animal Co., Ltd. These animals were housed in a controlled environment (22°C, 50–70% humidity, 12 h light/dark cycle) with free food and water access. Animal experiments were performed as per the guidance of the Institute of Animal Care and User Committee (IACUC). Animals were randomized into wild-type control (SAMR1 mice, NC), control (SAMP8 mice), ACP (SAMP8 mice treated with 25, 50, or 100 mg/kg/d ACP), and positive control (SAMP8 mice treated with 1.667 mg/kg/d donepezil HCl) groups.

### 2.6 Behavioral analyses

After a 7-week dosing period, murine cognitive function was assessed with the Morris water maze (MWM) test, using a slightly modified version of an approach reported previously (35). Briefly, mice were trained for 5 d using a circular basin (90 cm high, 100 cm in diameter) containing water at 22°C, with a hidden platform located 1 cm beneath the water surface. On each day of training, mice completed four trials, beginning each trial in a different quadrant. Mice were assessed to determine whether they reached the platform within 90 s, allowing the mouse to rest on the platform for 15 s after locating it prior to removing animals from the tank. When mice failed to reach the platform, they were manually guided to it and allowed to rest there for 15 s. After this 5-day training period, mice were placed opposite the location of the hidden platform, which was removed, and the number of platform crossings within 90 s as well as the proportion of time spent in that target quadrant were recorded with The Smart v3.0 Small Animal Behavioral Recording and Analysis System (Reward Corporation).

### 2.7 16S rDNA sequencing

An E.Z.N.A.^®^ soil DNA Kit (Omega Bio-Tek, GA, United States) was used to extract total genomic DNA from fecal samples as directed, after which DNA quality and concentration were measured using 1.0% agarose gel electrophoresis and a NanoDrop^®^ ND-2000 spectrophotometer (Thermo Scientific Inc., United States), followed by storage at-80°C. The 338F (5′-ACTCCTACGGGAGGCAGCAG-3′) and 806R (5′-GGACTACHVGGGTWTCTAAT-3′) primers targeting the V3–V4 hypervariable region of the 16S rRNA gene were used to amplify isolated DNA (36) with an ABI GeneAmp^®^ 9,700 PCR thermocycler (ABI, CA, United States). Each PCR reaction consisted of 4 *μ*L 5x Fast Pfu buffer, 2 μ: 2.5 mM dNTPs, 0.8 μL of each primer (5 μM), 0.4 μL of Fast Pfu DNA polymerase, 0.2 μL of BSA, 10 ng of template DNA, and ddH_2_O to 20 μL. Thermocycler settings were: 95°C for 3 min; 27 cycles of 95°C for 30 s, 55°C for 30 s, 72°C for 45 s; 72°C for 10 min, with a final resting incubation at 4°C. Sample amplification was conducted in triplicate, and PCR products were extracted following 2% agarose gel electrophoresis using an AxyPrep DNA Gel Extraction Kit (Axygen Biosciences, CA, United States) as directed, with quantification then being performed with a Quantus™ Fluorometer (Promega, United States).

### 2.8 Statistical analysis

Data are derived from three or more independent experiments and are presented as means ± standard deviation (x ± s). All analyses were performed using GraphPad Prism 8.0. When comparing data with a normal distribution and homogenous variance among multiple groups, one-way ANOVAs with the Student Newman–Keuls (SNK) multiple comparison test were used. *p* < 0.05 was regarded as the threshold for significance.

## 3 Results

### 3.1 ACP isolation, purification, and physicochemical characterization

**Figure 1:**
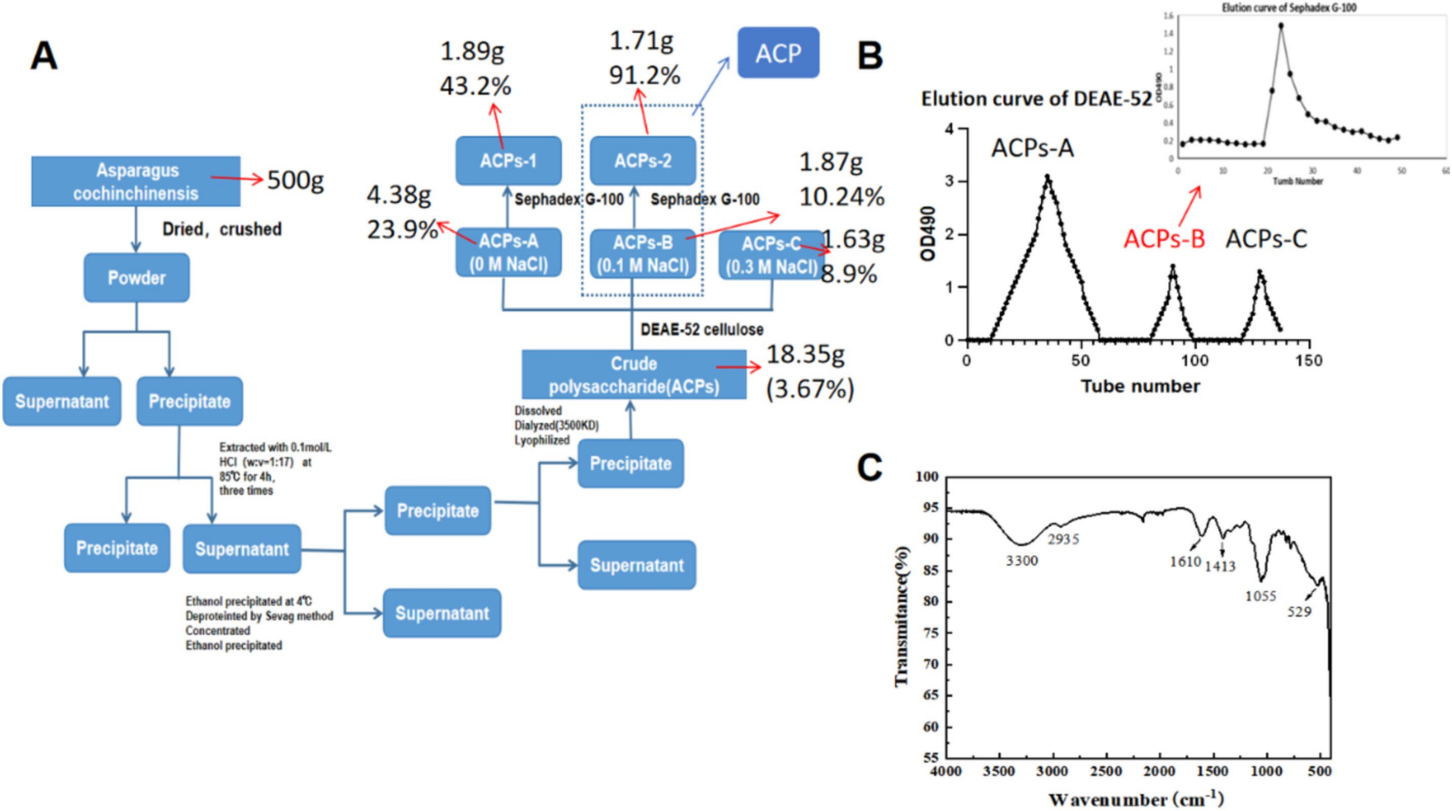
**(A)** Flow chart overview of the process of ACP purification. **(B)** ACP elution curves using a DEAE-52 column and a Sephadex G-100 column for the further purification of fraction. **(C)** ACP FT-IR spectrum.

Here, an acid-assisted extraction approach was used to isolate ACP, with a yield of 3.67% of the dry weight of the raw AC input material. Following ACP deproteinization and decolorization, samples were fractionated and purified with DEAE-52 and Sephadex G-200 chromatography columns (Figures 1A,B). Initial DEAE-52 column purification yielded three fractions, including deionized water, 0.1 mol/L NaCl, and 0.3 mol/L NaCl fractions with respective yields of 23.9, 10.24 and 8.9%. ACPs-B was subject to further Sephadex G-100 chromatography column purification, yielding a single symmetrical ACP peak with a 91.2% yield (not shown). In view of the previous product yield and the experimental effect in animals, we finally selected ACPs-2 (ACP). ACP had a calculated molecular weight of 15,580 Da, and its polydispersity index was ~1.14, with this value being close to 1 such that this ACP fraction was regarded as likely being homogenous (Table 1).

**Table 1:** ACP physicochemical properties.

	ACP
Yield (%)	3.67 ± 0.07^a^
Carbohydrate (%)	93.41 ± 3.95^c^
Protein (%)	ND
Polyphenols (%)	0.79 ± 0.01^a^
Molecular weight (Da)	
Weight-average molecular weight (Mw)	15,580 Da
Number-average molecular weight (Mn)	13,667 Da
Polymer dispersity index (PDI)	1.14

### 3.2 FT-IR analyses

Infrared spectral analyses are commonly used to detect functional groups including O–H, C–H, and C=O. ACP samples exhibited FT-IR signals at 3330, 2935, 1,413, and 1,055 cm^−1^ that are characteristic of polysaccharides (Figure 1C) (30). The peak at 3411 cm^−1^ was consistent with O–H hydrogen bond vibrations, while the peak at 2933 cm^−1^ was consistent with C–H vibrations, and the peaks at 3411 cm^−1^ and 2,933 cm^−1^ were indicative of the presence of a polysaccharide sample (24). Absorption bands in the 1,500–400 cm^−1^ region were sensitive to changes, with the peaks at 1036 cm^−1^ and 1,109 cm^−1^ confirming that C–O–C and C–O–H stretching vibrations were present, indicative of a pyranose ring. Signals were also observed at 529 cm^−1^ and 1,100–1,420 cm^−1^.

### 3.3 Analysis of ACP monosaccharide composition

ACP was found to primarily consist of glucose, galactose, L-fucose, and fructose at a 82.14:12.23:2.61:2.49 ratio, with trace levels of xylose, arabinose, and rhamnose at a 0.48:0.04:0.02 ratio (Table 2). In other reports, ACP was indicated to include fructose and glucose at a 93.3:6.7 molar ratio (37), with other studies having also described ACP preparations containing xylose, arabinose, glucose, rhamnose, mannose, galactose, glucuronic acid, and galacturonic acid (38). These differences in monosaccharide composition and molar ratios are likely related to differences in ACP sources or the methods employed for extraction and purification.

**Table 2:** ACP monosaccharide composition.

Monosaccharide composition	Molar ratio (%)
Arabinose	0.04
Glucose	82.14
Galactose	12.23
Xylose	0.48
Fructose	2.49
Rhamnose	0.02
Fucose	2.61

### 3.4 Analyses of ACP methylation

Methylation analyses can offer significant insight regarding polysaccharide structural characteristics. As it contained little uronic acid, the direct and complete methylation of all free OH groups in ACP was observed. Following the hydrolysis, reduction, and acetylation of permethylated polysaccharides to isolate PMAAs, these samples were subjected to GC–MS analysis (38). Peak areas in the GC chromatogram were compared to compute the molar percentage ratios for various sugar residue types, ultimately revealing the presence of Glc*p*-(1→, →2)-Glc*p*-(1→, →6)-Glc*p*-(1→, →4)-Glc*p*-(1→, →3, 4)-Glc*p*-(1→, →2,4) -Gal*p*-(1→, →4,6)-Gal*p*-(1→, and →3,4,6)-Gal*p*-(1 → sugar linkages in ACP at molar percent ratios of 23.70:1.30:3.55:50.77:6.91:1.10:11.50:1.18 (Table 3). Based on the monosaccharide and methylation linkage analysis results, ACP was primarily composed of glucose (50.77%), consistent with the →4)-Glc*p*-(1 → residues in the backbone of the ACP structure.

**Table 3:** ACP methylation analysis results.

Linkage patterns	Derivative name	RT	Relative molar ratio (%)
t-Glc(*p*)	1,5-di-O-acetyl-2,3,4,6-tetra-O-methyl glucitol	9.005	23.70
2-Glc(*p*)	1,2,5-tri-O-acetyl-3,4,6-tri-O-methyl glucitol	12.565	1.30
6-Glc(*p*)	1,5,6-tri-O-acetyl-2,3,4-tri-O-methyl glucitol	13.803	3.55
4-Glc(*p*)	1,4,5-tri-O-acetyl-2,3,6-tri-O-methyl glucitol	14.21	50.77
3,4-Glc(*p*)	1,3,4,5-tetra-O-acetyl-2,6-di-O-methyl glucitol	16.326	6.91
2,4-Gal(*p*)	1,2,4,5-tetra-O-acetyl-3,6-di-O-methyl galactitol	16.975	1.10
4,6-Gal(*p*)	1,4,5,6-tetra-O-acetyl-2,3-di-O-methyl galactitol	18.432	11.50
3,4,6-Gal(*p*)	1,4,5,6-tetra-O-acetyl-2,3-di-O-methyl galactitol	20.815	1.18

### 3.5 NMR analyses of ACP

**Figure 2:**
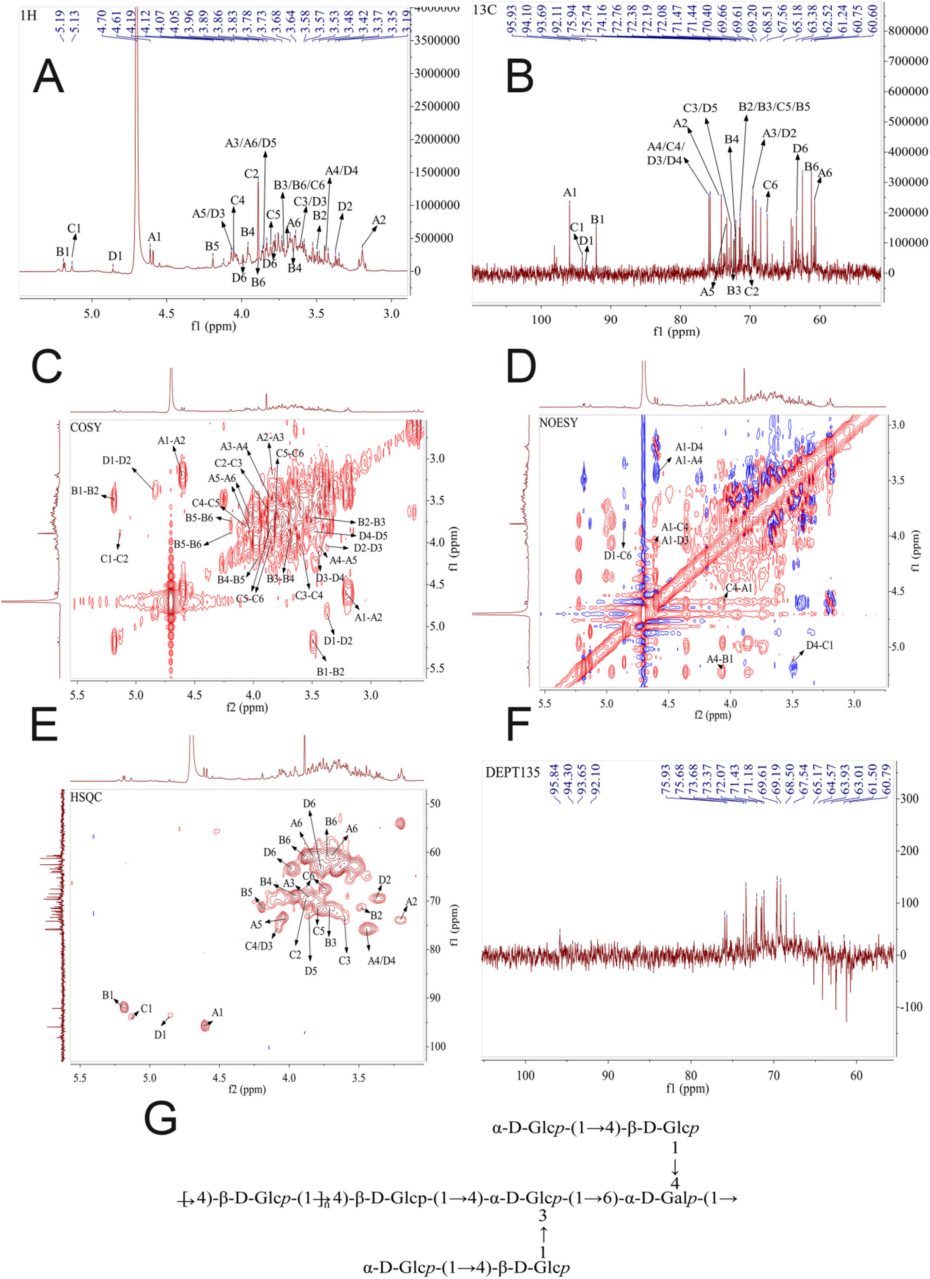
**(A)** ^1^H NMR spectrum for ACP. **(B)**^13^C NMR spectrum for ACP. **(C)** ^1^H-^1^H COSY spectrum for ACP. **(D)** NOESY spectrum for ACP. **(E)** 2D NMR spectra for ACP., with HSQC and HMBC overlays. **(F)** DEPT 135 NMR spectrum for ACP. **(G)** The possible structure of ACP.

An NMR approach was next used to gain further insight into the structural characteristics of ACP, including the glycosidic bond connections and configurations present therein. Virtually all protons in the ACP ^1^H NMR spectra wherein the 3.00–5.30 ppm range (Figure 2A), as is normal for polysaccharides. The strong signal peak at 4.70 ppm corresponds to the D_2_O solvent peak. Glycosidic bond configurations can be determined based on allocephalic hydrogenation shifts, with *α*-and *β*-configuration polysaccharide molecules generally exhibiting these shifts in the 5.0–5.5 ppm and 4.5–5.0 ppm ranges, respectively. The majority of ACP heterohydrogenation shifts fell in the 2.15–2.75 and 3.15–4.25 ppm ranges (Figure 2A). Heteropolytic proton signals at 4.50, 4.55, 4.59, 4.61, and 4.86 ppm were assigned to β-pyranose units, while signals at 5.13, 5.18, and 5.19 ppm were assigned to *α*-pyranose units. These data were consistent with large numbers of glycosidic bonds in the *β* configuration together with a relatively limited number in the α configuration in ACP.

Chemical shifts in the 1-dimensional carbon NMR spectrum were wider than those for the proton NMR spectrum, with slightly higher resolution (Figure 2B). These carbon spectral data can be employed to determine the positions and molecular conformation of groups. Polysaccharide heterocephalic carbon signals are generally observed in the *δ*_C_ 90–113 ppm range, with *α*-type glycosidic bonds exhibiting shifts < *δ*_C_ 102 ppm. ACP exhibited bonds in the *β*-configuration, with seven signal peaks in the δ_C_ 90–113 ppm range including signals at *δ*_C_ 92.10, 95.93, at 98.10 ppm, supporting the presence of both α-and β-type bonds within ACP. Furanose C3 and C5 signals were present in the δ 82–84 ppm range, while pyranose C3 and C5 signals were < 80 ppm, enabling differentiation between the two.

For residue A, anomeric hydrogen and carbon chemical shifts (4.61/95.93 ppm) were consistent with glucose in the β-configuration. In the COSY map, H2 (3.2 ppm) of residue A was determined based on cross peak 4.61/3.2 ppm, and H3 (3.85 ppm) of residue A was determined based on cross peak 3.2/3.85 ppm. H4 (3.45 ppm) of residue A was determined based on cross peak 3.85/3.45 ppm, and H5 (4.04 ppm) of residue A was determined based on cross peak 3.45/4.04 ppm. The H6 (3.83, 3.69 ppm) of residue A was determined from the cross peak 4.04/3.83, 3.69 ppm, which can be attributed to the chemical shift of hydrogen on the complete sugar ring. Then, the chemical shift of C on the sugar ring is attributed by HSQC signal. The chemical shift of C1 of residue A is 95.93 ppm, the chemical shift of C2 of residue A is 74.16 ppm, the chemical shift of C3 of residue A is 69.53 ppm, and the chemical shift of C4 of residue A is 75.66 ppm. The C5 chemical shift of residue A is 73.48 ppm, the C6 chemical shift of residue A is 60.75 ppm, and the chemical shift of C1 and C4 is shifted to the low field, indicating that the residue is replaced at the O-1, O-4 position of the sugar ring. Combined with the results of methylation analysis and literature reports, it is inferred that the residue A was concluded to correspond to →4)-*β*-D-Glc*p*-(1 →.

For residue B, anomeric hydrogen and carbon chemical shifts (5.19/92.11 ppm) were consistent with glucose in the *α*-configuration. In the COSY map, H2 (3.49 ppm) of residue B was determined based on cross peak 5.19/3.49 ppm, and H3 (3.7 ppm) of residue B was determined based on cross peak 3.49/3.7 ppm. H4 (3.95 ppm) of residue B was determined based on cross peak 3.7/3.95 ppm, and H5 (4.2 ppm) of residue B was determined based on cross peak 3.95/4.2 ppm. The H6 (3.88, 3.72 ppm) of residue B was determined from the cross peak 4.2/3.88, 3.72 ppm, which can be attributed to the chemical shift of hydrogen on the complete sugar ring. Then, the chemical shift of C on the sugar ring is attributed by HSQC signal. The chemical shift of C1 of residue B is 92.11 ppm, the chemical shift of C2 of residue B is 71.47 ppm, the chemical shift of C3 of residue B is 71.37 ppm, and the chemical shift of C4 of residue B is 69.27 ppm. The C5 chemical shift of residue B is 71.44 ppm, the C6 chemical shift of residue B is 61.24 ppm, and the chemical shift of C1 is shifted to the low field, indicating that the residue is replaced at the glycocyclic O-1 position. Combined with the results of methylation analysis and literature reports, it is inferred that the carbohydrate residue B may be *α*-D-Glc*p*-(1→).

For residue C, anomeric hydrogen and carbon chemical shifts (5.13/94.1 ppm) were consistent with galactose in the α-configuration. In the COSY map, H2 (3.89 ppm) of residue C was determined according to the cross peak 5.13/3.89 ppm, and H3 (3.6 ppm) of residue C was determined according to the cross peak 3.89/3.6 ppm. H4 (4.06 ppm) of residue C was determined according to the cross peak 3.6/4.06 ppm, H5 (3.8 ppm) of residue C was determined according to the cross peak 4.06/3.8 ppm, and H5 (3.8 ppm) of residue C was determined according to the cross peak 3.8/3.74. 3.88 ppm determines the H6 of residue C (3.74, 3.88 ppm), which can be attributed to the chemical shift of hydrogen on the complete sugar ring. Then, the chemical shift of C on the sugar ring is attributed by HSQC signal. The chemical shift of C1 on residue C is 94.1 ppm, the chemical shift of C2 on residue C is 70.4 ppm, the chemical shift of C3 on residue C is 72.76 ppm, and the chemical shift of C4 on residue C is 75.94 ppm. The C5 chemical shift of residue C is 71.52 ppm, and the C6 chemical shift of residue C is 67.56 ppm. The chemical shifts of C1, C4, and C6 are shifted to the lower field, indicating that the residue is replaced at the positions of O-1, O-4, and O-6 in the sugar ring. Combined with the results of methylation analysis and literature reports, it is inferred that the sugar residue C may be In the COSY map, H2 (3.89 ppm) of residue C was determined according to the cross peak 5.13/3.89 ppm, and H3 (3.6 ppm) of residue C was determined according to the cross peak 3.89/3.6 ppm. H4 (4.06 ppm) of residue C was determined according to the cross peak 3.6/4.06 ppm, H5 (3.8 ppm) of residue C was determined according to the cross peak 4.06/3.8 ppm, and H5 (3.8 ppm) of residue C was determined according to the cross peak 3.8/3.74. 3.88 ppm determines the H6 of residue C (3.74, 3.88 ppm), which can be attributed to the chemical shift of hydrogen on the complete sugar ring. Then, the chemical shift of C on the sugar ring is attributed by HSQC signal. The chemical shift of C1 on residue C is 94.1 ppm, the chemical shift of C2 on residue C is 70.4 ppm, the chemical shift of C3 on residue C is 72.76 ppm, and the chemical shift of C4 on residue C is 75.94 ppm. The C5 chemical shift of residue C is 71.52 ppm, and the C6 chemical shift of residue C is 67.56 ppm. The chemical shifts of C1, C4, and C6 are shifted to the lower field, indicating that the residue is replaced at the positions of O-1, O-4, and O-6 in the sugar ring. Combined with the results of methylation analysis and literature reports, it is inferred that the sugar residue C may be →4,6)-*α*-d-Gal*p* -(1 → .

For residue D, anomeric hydrogen and carbon chemical shifts (4.86/93.69 ppm) were consistent with glucose in the α-configuration. In the COSY map, H2 (3.37 ppm) of residue D was determined based on cross peak 4.86/3.37 ppm, and H3 (4.07 ppm) of residue D was determined based on cross peak 3.37/4.07 ppm. H4 (3.48 ppm) of residue D was determined based on cross peak 4.07/3.48 ppm, and H5 (3.86 ppm) of residue D was determined based on cross peak 3.48/3.86 ppm. The H6 (3.98, 3.77 ppm) of residue D was determined from the cross peak 3.86/3.98, 3.77 ppm, which can be attributed to the chemical shift of hydrogen on the complete sugar ring. Then, the chemical shift of C on the sugar ring is attributed by HSQC signal. The chemical shift of C1 of residue D is 93.69 ppm, the chemical shift of C2 of residue D is 69.66 ppm, the chemical shift of C3 of residue D is 75.66 ppm, and the chemical shift of C4 of residue D is 75.94 ppm. The C5 chemical shift of residue D is 72.77 ppm, and the C6 chemical shift of residue D is 63.38 ppm. The chemical shifts of C1, C3, and C4 are shifted to the low field, indicating that the residue is replaced at the positions of O-1, O-3, and O-4 in the sugar ring. Combined with the results of methylation analysis and literature reports, it is inferred that the sugar residue D may be →3,4)-*α*-D-Glc*p*-(1 → .

Based on these results from one-dimensional NMR (^1^H and ^13^C) and two-dimensional NMR (HSQC and HMBC) approaches, the fine structure of ACP was ultimately determined, as shown in Figure 2 and Table 4.

**Table 4:** ^1^H and ^13^C chemical shifts (ppm) in ACP.

Code	Glycosyl residues	Chemical shifts (ppm)					
		H1/C1	H2/C2	H3/C3	H4/C4	H5/C5	H6a,6b/C6
A	→4)-β-D-Glc*p*-(1→	4.61	3.2	3.85	3.45	4.04	3.83, 3.69
		95.93	74.16	69.53	75.66	73.48	60.75
B	α-D-Glc*p*-(1→	5.19	3.49	3.7	3.95	4.2	3.88, 3.72
		92.11	71.47	71.37	69.27	71.44	61.24
C	→4,6)-α-D-Gal*p*-(1→	5.13	3.89	3.6	4.06	3.8	3.74, 3.88
		94.1	70.4	72.76	75.94	71.52	67.56
D	→3,4)-α-D-Glc*p*-(1→	4.86	3.37	4.07	3.48	3.86	3.98, 3.77
		93.69	69.66	75.66	75.94	72.77	63.38

To further assess the structure of the backbone of ACP, 1D-and 2D-NMR spectra were analyzed, assigning ^1^H and ^13^C NMR signals based on correlations in the HMBC and NOESY spectra and values that have been reported in the literature. The HMBC and NOESY spectra can effectively reveal glycosylic linkages between sugar residues, and they can also reveal intra-residue connections, as shown in ACP. The NOESY spectrum was used to analyze connections in ACP, as it exhibited lower signal intensity at the cross peaks of the HMBC spectrum. Some cross peaks were observed in the NOESY spectrum (Figure 2D), including peaks corresponding to H1 of sugar residue A has A cross peak 4.61/3.45 ppm with H4 of sugar residue A, the H1 of sugar residue A has a cross peak 4.61/4.36 ppm with H4 of sugar residue E, and the H1 of sugar residue A has a cross peak 4.61/4.07 ppm with H3 of sugar residue D. The H1 of sugar residue B has A cross peak 5.19/3.45 ppm with H4 of sugar residue A, the H1 of sugar residue C has a cross peak 5.13/3.45 ppm with H4 of sugar residue A, and the H1 of sugar residue D has a cross peak 4.86/3.88 ppm with H6 of sugar residue C.

Therefore, based on one-dimensional and two-dimensional NMR information and methylation analysis, it is concluded that the polysaccharide is mainly composed of →4)-*β*-D-Glc*p*-(1 → and a small amount→4,6)-*α*-D-Gal*p*-(1 → and →3,4)-α-D-Glc*p*-(1 → and so on. Branched chain is mainly composed of α-D-Glc*p*-(1 → 4)-β-D-Glc*p*-(1 → connected to the sugar residues α-D-Glc*p*-(1 → 4)-β-D-Glc*p*-(1 → O-4 position or sugar residues of α-D-Glc*p*-(1 → 4)-β-D-Glc*p*-(1 → O-3 position.

### 3.6 SEM analyses

**Figure 3:**
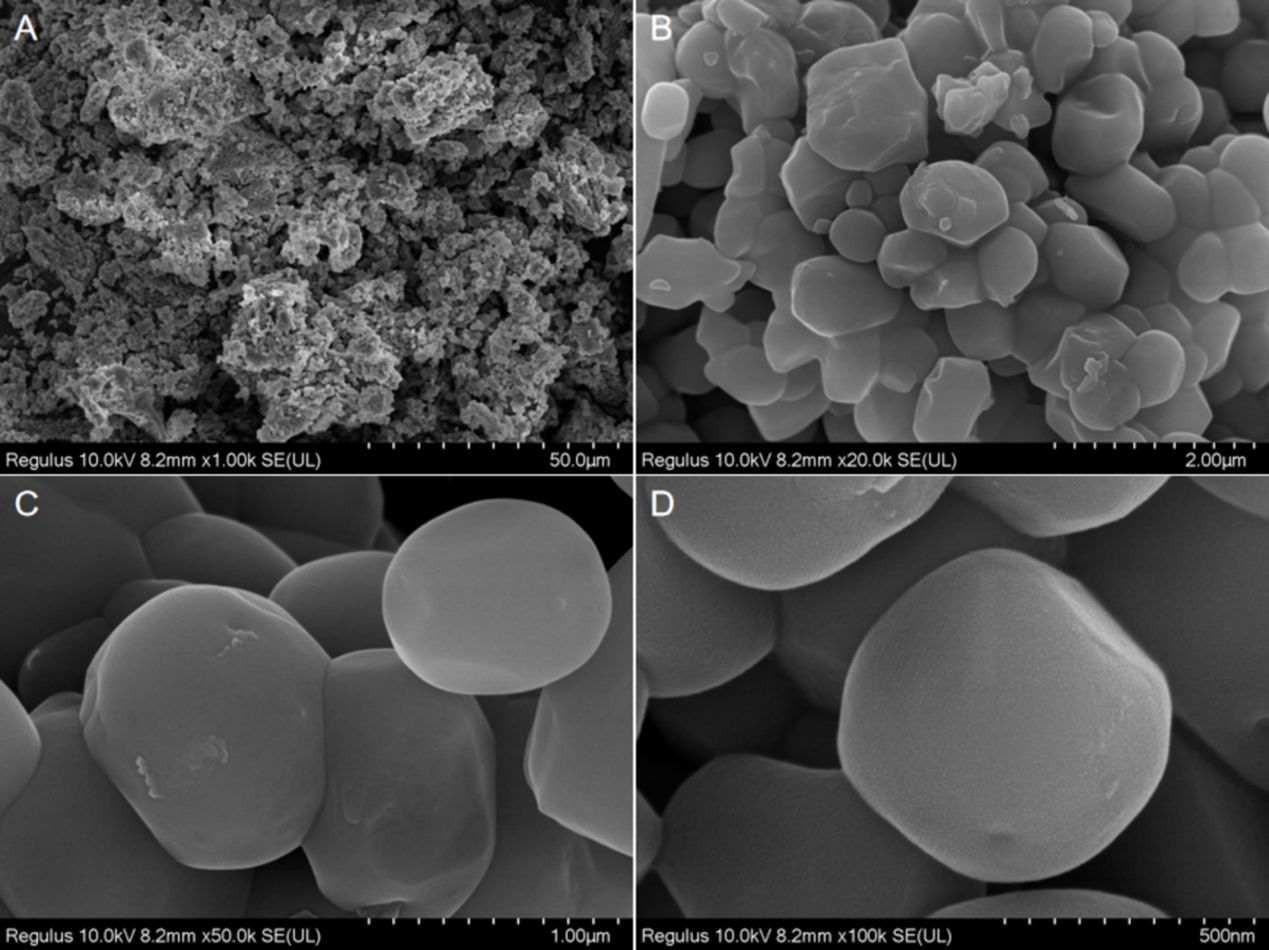
SEM images of the ACP at different multiples [1.00 k **(A)**, 20.0 k **(B)**, 50.0 k **(C)**, and 100 k **(D)**].

SEM approaches are often employed to assess the surfaces and microstructural characteristics of polysaccharides, offering insight into macromolecule morphology, shape, size, and porosity (39). SEM imaging revealed that all ACP samples presented with block-like structures after acid and water treatment (Figure 3). This may be attributable to cavitation activity, turbulence shearing, and instantaneous high pressures. Acid-assisted extraction can also disrupt cellular structures, increasing the contact area between the liquid and raw material phases. This may explain the block-like surface characteristics of ACP. Indeed, extraction and purification strategies have been confirmed to influence polysaccharide shape and surface topological characteristics.

### 3.7 ACP enhances the cognitive function of SAMP8 mice

**Figure 4:**
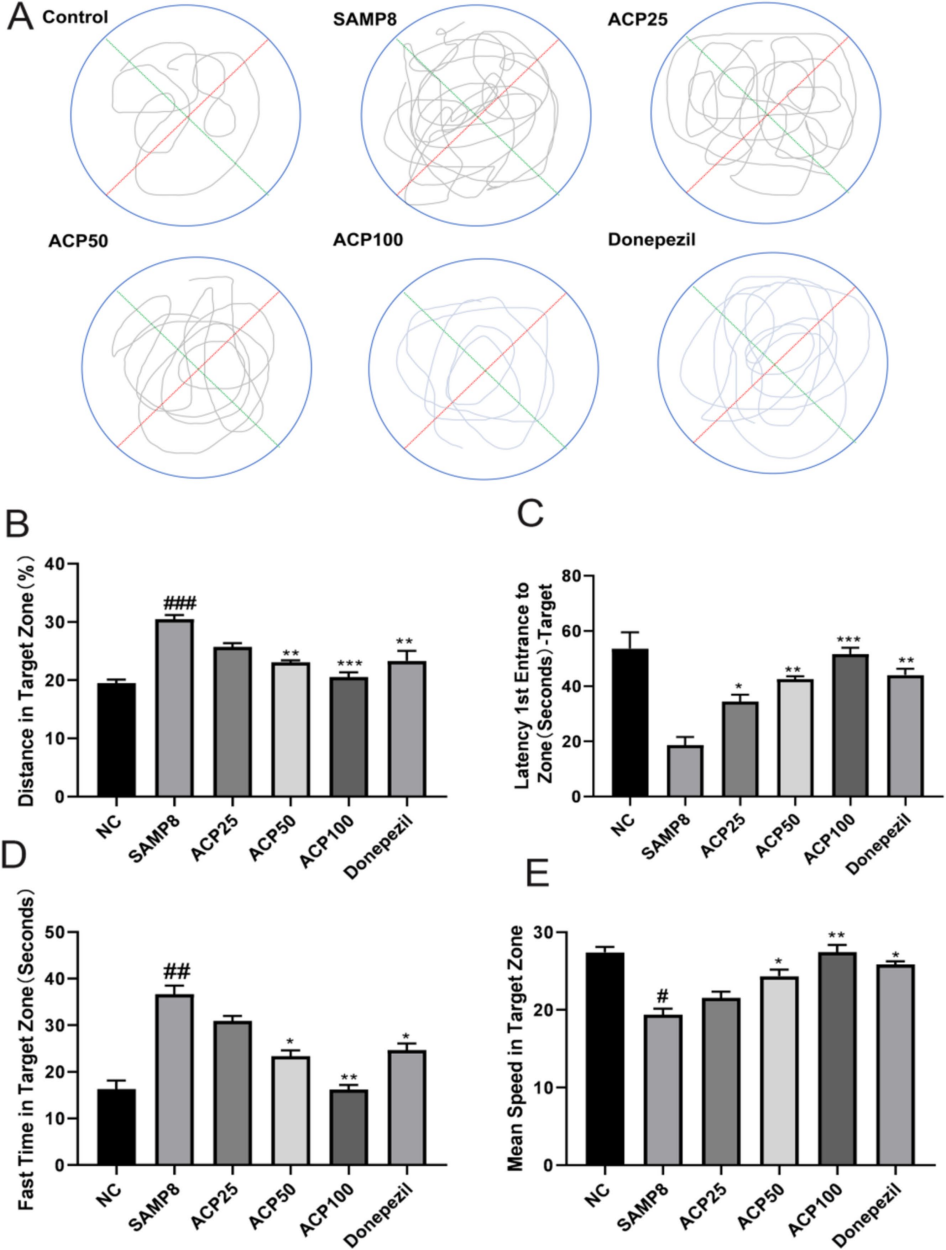
ACP treatment restores spatial learning and memory in SAMP8 mice. **(A)** Representative MWM traces for mice in the indicated groups. **(B)** Distance in target zone (%). **(C)** Latency 1st entrance to zone (s) -target. **(D)** Fast time in target zone (s). **(E)** Mean speed in target zone. (^#^ *p* < 0.05, ^###^ *p* < 0.001 vs. NC group; * *p* < 0.05, ** *p* < 0.01, *** *p* < 0.001 vs. SAMP8 group, *n* = 6).

The impact of ACP administration on spatial memory in SAMP8 model mice was assessed with the MWM, monitoring the swimming paths of mice during testing (Figure 4A). Relative to the NC group, SAMP8 model mice exhibited fewer platform crossings, while tighter paths and more platform crossings were observed for mice in the ACP50 and ACP100 groups as compared to the SAMP8 group. A similar improvement was also evident in the donepezil group. Compared to NC controls, SAMP8 mice also exhibited significantly increased Target Zone (%) and Fast Time in the Target Zone (s) values together with decreased Mean Speed in Target Zone and Latency 1st Entrance to Zone (s)-Target values, consistent with impaired spatial learning and memory (Figures 4B–E). A significant increase in the Latency 1st Entrance to Zone (s)-Target was also observed in the ACP25 group (*p* < 0.05), while significantly increased Distance in Target Zone (%) (*p* < 0.001) and Fast Time in Target Zone (s) (*p* < 0.05) were observed for the ACP50 group.

### 3.8 The impact of ACP on the composition of the gut microbiota in SAMP8 mice

#### 3.8.1 ACP promotes SCFA production

**Figure 5:**
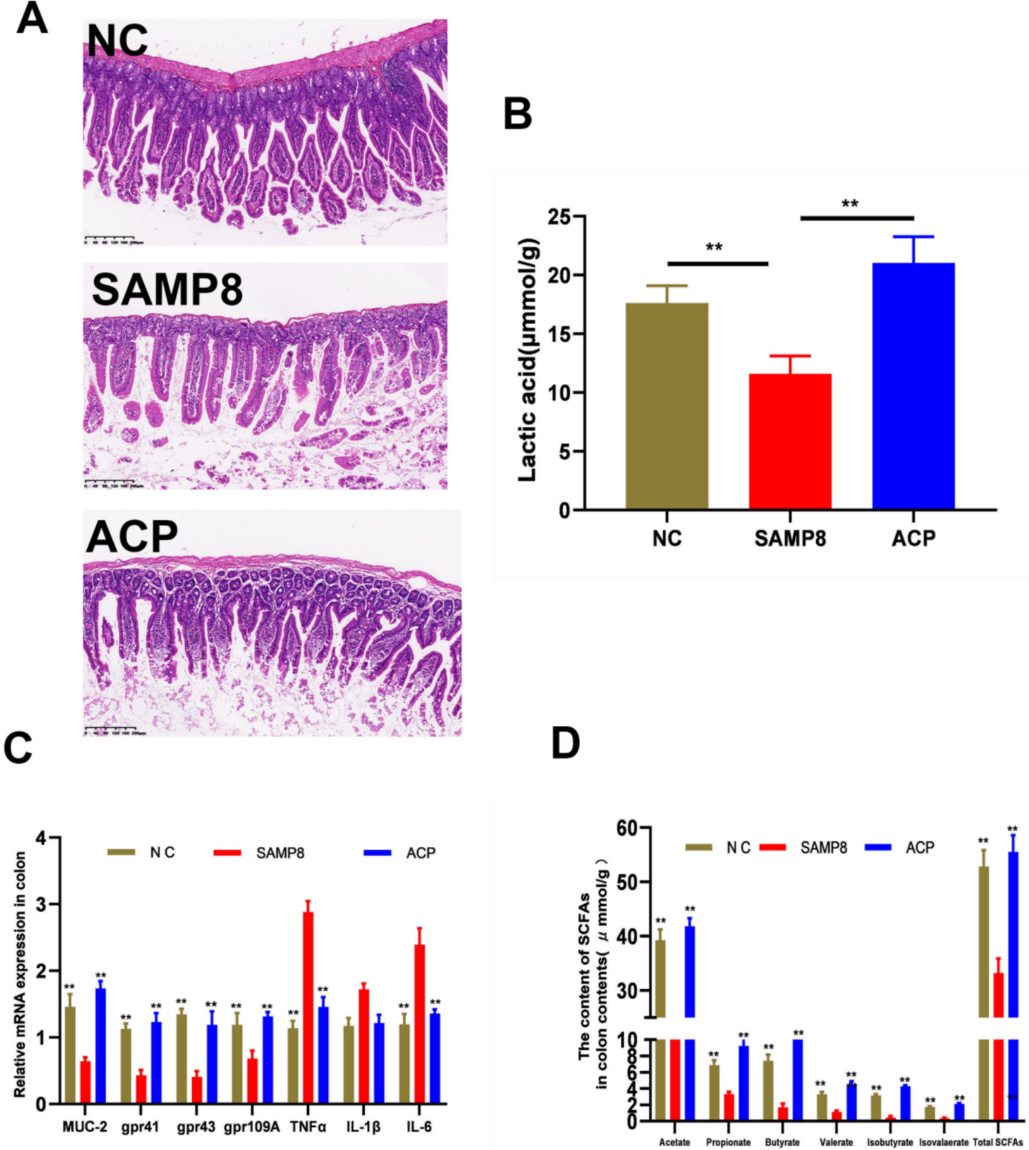
ACP alleviates intestinal barrier dysfunction and enhances the production of SCFAs in SAMP8 mice. **(A)** Representative H&E stained images of colonic sections from the indicated groups (Scale bar: 200 μm). **(B)** SCFA content in the colon contents. **(C)** Relative mRNA expression in the colon. **(D)** Lactic acid levels in colon contents. * *p* < 0.05, ** *p* < 0.01 vs. the SAMP8 group.

The SAMP8 can result in the dysfunction of the epithelial barrier owing to an increase in the permeability of the intestines. The impact of ACP on intestinal barrier integrity in SAMP8 mice was assessed based on intestinal morphology and the expression of TNF-*α*, MUC-2, and SCFA receptors. Representative H&E stained sections of colon tissue are presented in Figure 5A, revealing clear crypt structures and the absence of inflammatory infiltration or damage in mice from the NC group. In contrast, pronounced mononuclear cell infiltration and crypt deformities were evident in SAMP8 mice, whereas ACP administration reversed these effects, consistent with the ability of such treatment to abrogate chronic inflammation and enhance the integrity of the epithelial barrier in the colon of these SAMP8 mice. Significantly reduced TNF-*α* expression was also detected in the colon of ACP-treated mice relative to SAMP8 model controls (Figure 5C). Goblet cells produce the mucin MUC-2, and ACP treatment significantly lowered these *Muc2* mRNA levels relative to the SAMP8 group. ACP is thus capable of augmenting intestinal epithelial integrity through the enhanced secretion of mucins and the suppression of TNF-α secretion into the systemic circulation.

Short-chain fatty acids (SCFAs) are the primary polysaccharide metabolites within the intestines. Levels of acetic, propionic, n-butyric, and i-valeric acids in the colon contents of model mice were significantly reduced relative to those in samples from NC mice, whereas ACP administration significantly reversed this change, consistent with the ability of ACP to promote CFA production within the colon of SAMP8 mice (Figure 5B). Moreover, ACP administration significantly enhanced *Gpr43, Gpr41,* and *Gpr109A* expression relative to the SAMP8 group (Figure 5C). ACP also significantly increased lactic acid levels in the colon contents from these mice (Figure 5D). Together these data support the ability of ACP to increase SCFA production, thereby promoting GPR upregulation within the colon.

#### 3.8.2 ACP modulates gut microbiota composition and functionality in SAMP8 mice

The aging process has been linked to both gastric dysfunction and degenerative changes in the nervous system that can contribute to gastrointestinal dysbiosis reflected by the impairment of the makeup and function of the gut microbiota (40). The composition of the gut microflora can also reportedly affect the rate of aging (41), with dysbiosis being closely related to AD incidence and progression. These gut microbes can engage in communication with the central nervous system via endocrine, immunological, and neural pathways, potentially contributing to the pathogenesis of neurodegeneration through the production of deleterious compounds, the regulation or secretion of neurotransmitters, and the induction of neuroinflammation.

**Figure 6:**
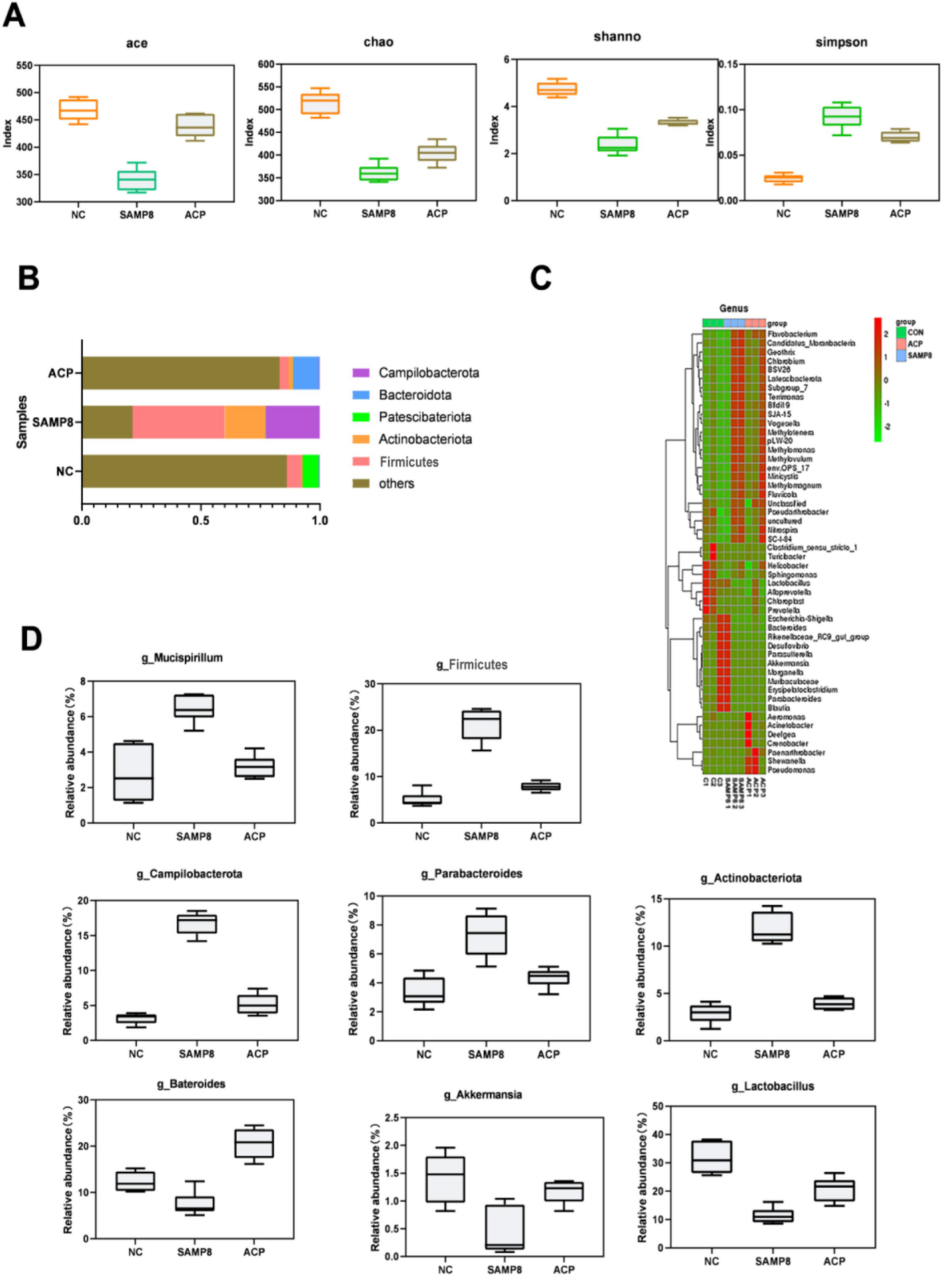
The impact of ACP on the gut microbiota in SAMP8 mice. **(A)** Ace, Chao1, Shannon, and Simpson index values were used to assess alpha diversity. **(B)** Relative gut microbe abundance at the phylum level. **(C)** Relative gut microbe abundance at the genus level. **(D)** Relative *Mucispirillum, Actinobacteriota, Firmicutes, Akkermansia, Bateroides, Lactobacillus,* and *Campilbacterota* abundance.

In this study, a 16S rDNA sequencing approach was used to evaluate changes in the gut microflora of analyzed mice. Based on the results of behavioral and oxidative stress analyses, ACP100 treatment yielded therapeutic efficacy superior to that of ACP50 and ACP25. Accordingly, the ACP100 dose was selected for use in these experiments exploring the pharmacodynamic effects of ACP in an effort to better understand its anti-aging mechanisms. Sequencing of the V3–V4 hypervariable region in fecal DNA samples from mice in the NC, SAMP8, and ACP treatment groups was conducted, after which the Ace, Chao1, Shannon, and Simpson indices were used to evaluate microbial alpha diversity, revealing pronounced differences in the microbial species present in these samples among groups (Figure 6A). Relative to the NC group, a significant reduction in gut microbiota diversity was evident in SAMP8 mice, while this diversity was restored with ACP treatment.

Further analyses of the gut microbiota in these mice were conducted at the phylum and genus levels. Dominant phyla in NC mice included *Bacteroidota, Actinobacteriota, Patescibacteria, Firmicutes,* and *Campilobacterota*, with the same composition being evident in other groups. A change in the gut microbiota composition was evident in the SAMP8 mice. A significant change in the aging-related B/F (*Bacteroidota/Firmicutes*) ratio was also evident, with respective values of 1.51, 0.52, and 1.74 in the NC, SAMP8, and ACP treatment groups. At the genus level, SAMP8 mice exhibited increases in the proportions of *Mucispirillum, Actinobacteriota, Firmicutes,* and *Campilbacterota* together with reductions in the proportions of *Bacteroides, Akkermansia,* and *Lactobacillus*. Following ACP treatment, these changes were reversed (Figures 6B–D).

## 4 Discussion and conclusion

AD is a form of chronic neurodegenerative disease with a complex and incompletely understood pathological basis such that there is a pressing need to explore novel approaches to treating affected patients (42, 43). Many different processes and signaling pathways are involved in AD, with clear roles for inflammation, apoptosis, and oxidative stress in this setting (4). The dysbiosis of the gut microflora can also impact hippocampal Aβ clearance in AD patients, further potentiating disease development (44). Mechanistically, this loss of intestinal homeostasis can compromise the integrity of the intestinal barrier, resulting in the extravasation of inflammatory mediators that ultimately trigger or exacerbate inflammatory disease-related processes. Changes in the structural composition of the gut microflora have been shown to be associated with direct or indirect changes in neurotransmitter levels and the production of bacterial metabolites, which serve as signaling intermediaries between the gut and the brain. Through this pathway, but microbes can influence host biochemical and neurophysiological processes, and can modulate neuroinflammation in the brain via disrupting blood–brain barrier integrity. These processes ultimately result in altered brain function and behavior, and can contribute to the pathogenesis of AD. Changes in intestinal flora abundance and/or function can result in damage to the intestinal tissue, disrupting intestinal mucosal stability and integrity while triggering a range of inflammatory responses. The release of gut microbe-derived metabolites including SCFAs and 5-HT can also trigger depressive symptoms in the brain, which can also occur as a result of the effects of these microbes on the hypothalamic–pituitary–adrenal axis. Plant polysaccharides can serve as a form of prebiotic that can be used by intestinal microbes so as to stimulate the growth of beneficial bacteria, thereby potentially modulating the development of AD via the microbiota-gut-brain axis.

Here, ACP was identified as a novel polysaccharide extracted from *A. cochinchinensis* that subsequently underwent structural characterization and analyses of its *in vivo* anti-AD effects. Structural characteristics can be used to categorize polysaccharides as glucans, mannoglucans, fructans, pectins, galactans, and arabogalactans. An inulin-type fructan with a molecular weight of 2,690 Da denoted AC neutral polysaccharide was previously isolated from *A. cochinchinensis* (Lour.). One-dimensional NMR, two-dimensional NMR, and methylation analyses ultimately revealed that the polysaccharide is mainly composed of →4)-*β*-D-Glcp-(1 → and a small amount→4,6)-*α*-D-Galp-(1 → and →3,4)-α-D-Glcp-(1 → and so on. Branched chain is mainly composed of α-D-Glcp-(1 → 4)-β-D-Glcp-(1 → connected to the sugar residues α-D-Glcp-(1 → 4)-β-D-Glcp-(1 → O-4 position or sugar residues of α-D-Glcp-(1 → 4)-β-D-Glcp-(1 → O-3 position. These assays revealed the ability of ACP to protect against intestinal dysbiosis and cognitive impairment in AD model mice. This polysaccharide was able to enhance learning and memory in these SAMP8 mice while also mitigating oxidative stress within the brain. Prior research suggests that gastrointestinal dysbiosis can trigger innate immune activity that results in mild chronic inflammation, which contributes to age-related degenerative processes, cognitive impairment, and the aging process as a whole. With more advanced age, gut microbiota diversity also declines, with accompanying reductions in Bifidobacteria levels and Firmicutes and Proteobacteria enrichment (45). In this study, the gut microbiota of SAMP8 exhibited disturbances with respect to the abundance of *Bacteroidota, Actinobacteriota, Patescibacteria, Firmicutes,* and *Campilobacterota*. Aging is also associated with an increase in the F/B ratio, influencing cognitive impairment and oxidative stress in the aging context (46). ACP-treated mice exhibited the reversal of these changes, suggesting that ACP was capable of alleviating oxidative stress and overcoming learning and memory deficits in part via modulating the gut microbiota composition in these SAMP8 mice.

## 5 Conclusion

In summary, A polysaccharides extracted from *A. cochinchinensis* can protect against Alzheimer’s disease by regulating the microbiota-gut-brain axis. This polysaccharide was mainly composed with glucans, mannoglucans, fructans, pectins, galactans, and arabogalactans. Its molecular weight was 15,580 Da. The main chain is mainly composed of →4)-*β*-D-Glc*p*-(1 → and a small amount→4,6)-*α*-D-Gal*p*-(1 → and →3,4)-α-D-Glc*p*-(1 → and so on. The animal experiments have shown that ACP was able to enhance learning and memory in these SAMP8 mice while also mitigating oxidative stress within the brain. ACP also reversed the interference of *Bacteroidota, Actinobacteriota, Patescibacteria, Firmicutes,* and *Campilobacterota* in SAMP 8 mice. Therefore, ACP has the potential to prevent Alzheimer’s disease.

## Data Availability

The original contributions presented in the study are included in the article/supplementary material, further inquiries can be directed to the corresponding authors.
